# Supplementary material for: Targeted Metabolomics to Assess Exposure to Environmental Chemicals of Concern in Japanese Quail at Two Life Stages
Source: Metabolites. 2021 Dec 8;11(12):850. doi: 10.3390/metabo11120850 (PMC8715744; doi:10.3390/metabo11120850)
Supplement: Supplementary file 1 [file metabolites-11-00850-s001.zip › REVISED Supplementary files EL/Supplemental figures.pdf]

Supplemental figures

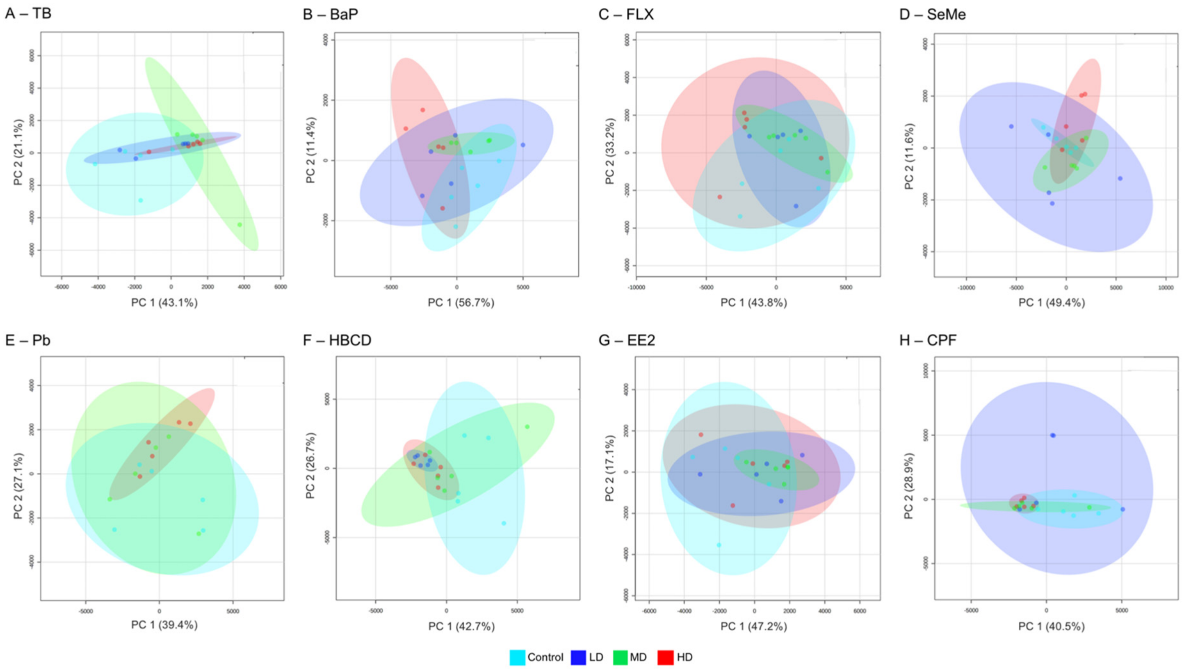

Figure S1: Principal Component Analysis (PCA) scores plot of the adult Japanese quail (JQ) liver metabolome following exposure to trenbolone (TB; A), benzo[a]pyrene (BaP; B), fluoxetine (FLX; C), seleno-L-methionine (SeMe; D), lead(II)nitrate (Pb, E), hexabromocyclododecane (HBCD; F), ethinylestradiol (EE2; G) and chlorpyrifos (CPF; H). JQ were exposed to three doses of each chemical defined as: low (LD, dark blue), medium (MD, green) and high (HD, red).
